# Supplementary material for: Clathrin light chain A drives selective myosin VI recruitment to clathrin-coated pits under membrane tension
Source: Nat Commun. 2019 Oct 31;10:4974. doi: 10.1038/s41467-019-12855-6 (PMC6823378; doi:10.1038/s41467-019-12855-6)
Supplement: Supplementary file 1 — Reporting Summary [file 41467_2019_12855_MOESM1_ESM.pdf]

nature research

Corresponding author(s): NCOMMS-19-17553C

Last updated by author(s): 2019/09/27

Reporting Summary

Nature Research wishes to improve the reproducibility of the work that we publish. This form provides structure for consistency and transparency in reporting. For further information on Nature Research policies, see Authors & Referees and the Editorial Policy Checklist .

Statistics

For all statistical analyses, confirm that the following items are present in the figure legend, table legend, main text, or Methods section.

n/a	Confirmed	
☐	☒	The exact sample size (*n*) for each experimental group/condition, given as a discrete number and unit of measurement
☐	☒	A statement on whether measurements were taken from distinct samples or whether the same sample was measured repeatedly
☐	☒	The statistical test(s) used AND whether they are one- or two-sided<br>*Only common tests should be described solely by name; describe more complex techniques in the Methods section.*
☒	☐	A description of all covariates tested
☐	☒	A description of any assumptions or corrections, such as tests of normality and adjustment for multiple comparisons
☐	☒	A full description of the statistical parameters including central tendency (e.g. means) or other basic estimates (e.g. regression coefficient) AND variation (e.g. standard deviation) or associated estimates of uncertainty (e.g. confidence intervals)
☒	☐	For null hypothesis testing, the test statistic (e.g. *F*, *t*, *r*) with confidence intervals, effect sizes, degrees of freedom and *P* value noted<br>*Give P values as exact values whenever suitable.*
☒	☐	For Bayesian analysis, information on the choice of priors and Markov chain Monte Carlo settings
☒	☐	For hierarchical and complex designs, identification of the appropriate level for tests and full reporting of outcomes
☐	☒	Estimates of effect sizes (e.g. Cohen's *d*, Pearson's *r*), indicating how they were calculated

Our web collection on statistics for biologists contains articles on many of the points above.

Software and code

Policy information about availability of computer code

Data collection	ITC (Isothermal titration calorimetry) measurements were performed on a MicroCal PEAQ-ITC (Malvern Panalytical, Malvern, UK) instrument. Raw data were analyzed with the integrated Malvern analysis software, and heat production was fitted to a one-set-of-sites binding model.<br>For Negative staining of clathrin cages, images were obtained using a SIS Morada digital camera and TIA software (FEI).<br>For NMR data, the structure calculations were performed with Xplor-NIH (version 2.47). Pymol (Schrödinger) was used for visualization and figure generation; MOLMOL was used for r.m.s.d. calculation and figure generation.
Data analysis	All statistical analyses were performed using GraphPad Prism.

For manuscripts utilizing custom algorithms or software that are central to the research but not yet described in published literature, software must be made available to editors/reviewers. We strongly encourage code deposition in a community repository (e.g. GitHub). See the Nature Research guidelines for submitting code & software for further information.

Data

Policy information about availability of data

All manuscripts must include a data availability statement. This statement should provide the following information, where applicable:

- Accession codes, unique identifiers, or web links for publicly available datasets
- A list of figures that have associated raw data
- A description of any restrictions on data availability

All uncropped immunoblots associated with Figs. 1b, 1c, 1d, 1e, 2b,4f, 4d, 5a, 6b, 6c and Supplementary Figs. 1a, 1c, 2d, 9a, 9b, 10a,10c, 11c, 11d, 11e,12a, 12b are provided as Source Data file together with the Source Data for Figs. 2c and 4f.

Chemical shift assignments for the complex of myosin VI1050-1131 and CLCa46-61 have been deposited in the Biological Magnetic Resonance Bank under the ID

code 30500, and the atomic coordinates for the complex have been deposited in the Protein Data Bank under the ID code 6E5N.

Field-specific reporting

Please select the one below that is the best fit for your research. If you are not sure, read the appropriate sections before making your selection.

☒ Life sciences ☐ Behavioural & social sciences ☐ Ecological, evolutionary & environmental sciences

For a reference copy of the document with all sections, see nature.com/documents/nr-reporting-summary-flat.pdf

Life sciences study design

All studies must disclose on these points even when the disclosure is negative.

Sample size	No sample size calculation was performed.
Data exclusions	No data was excluded from the analysis.
Replication	All experiments were successfully reproduced (excluding occasional failures due to technical errors).
Randomization	Not relevant for our study.
Blinding	Researchers were blinded to group allocation during immunofluorescence, EM data collection

Reporting for specific materials, systems and methods

We require information from authors about some types of materials, experimental systems and methods used in many studies. Here, indicate whether each material, system or method listed is relevant to your study. If you are not sure if a list item applies to your research, read the appropriate section before selecting a response.

Materials & experimental systems

n/a	Involved in the study	
☐	☒	Antibodies
☐	☒	Eukaryotic cell lines
☒	☐	Palaeontology
☒	☐	Animals and other organisms
☒	☐	Human research participants
☒	☐	Clinical data

Methods

n/a	Involved in the study	
☒	☐	ChIP-seq
☒	☐	Flow cytometry
☒	☐	MRI-based neuroimaging

Antibodies

Antibodies used

Antibody/ Species/ Supplier /Code/ WB dilution /IF dilution
anti-GFP Rabbit Sigma G1544 1:5000 1:200
RFP-TRAP_A Llama Chromotek rta-20
anti-myosin VI Rabbit Ref. 1
1296 1:2000 1:400 -1:200*
anti-CHC, clone X22 Mouse Pierce MA1-065 1:2000
anti-CHC, clone 23 Mouse BD bioscience 610499 1:1000
anti-CLCa Mouse Ref. 2
X16 1:500
anti-CLCa, CLTA Rabbit Proteintech 10852-1-AP 1:500
anti-CLCb, CLTB Rabbit Proteintech 10455-1-AP 1:2000
anti-Dab2 Mouse BD bioscience 610464 1:2000
anti-Hip1R Rabbit Sigma HPA038135 1:1000
anti-actin Mouse Sigma 1:1000
anti-tubulin Mouse Sigma 1:1000
anti-E-cadherin Mouse BD Biosciences 610181 1:100*
anti-Zo-1 Rabbit Thermo Fisher 40-2200 1:100*
anti-Occludin Mouse SCBT E5 1:100*
Anti-PKC zeta Rabbit Santa Cruz Sc-216 1:100*

anti-His Mouse GE Healthcare 27-4710-01 1:1000
phalloidin TRITC 1:50
anti-Mouse IgG HRP Goat Bio-Rad 1721011 1:10000
anti-Rabbit IgG HRP Goat Bio-Rad 1706515 1:10000
anti-rabbit Alexa647 Donkey Thermo Fischer A31571 1:400-1:100*
anti-rabbit Alexa488 Donkey Thermo Fischer A21206 1:400-1:100*
anti-mouse Alexa488 Donkey Thermo Fischer A21202 1:400-1:100*
anti-rabbit Cy3 Donkey Jackson Lab 711-165-152 1:800
anti-mouse Cy3 Donkey Jackson Lab 715-165-150 1:800-1:100*
*the indicated dilution was used for immunofluorescence of Cysts.

Validation

Where possible, the antibodies were validated for western blot by using RNAi depleted cells as negative controls.
Reference for antibodies:
Ref. 1 Wollscheid, H. P. et al. Diverse functions of myosin VI elucidated by an isoform-specific alpha-helix domain. Nat Struct Mol Biol 23, 300-308, doi:10.1038/nsmb.3187 (2016).
Ref. 2 Brodsky, F. M. Clathrin structure characterized with monoclonal antibodies. I. Analysis of multiple antigenic sites. The Journal of cell biology 101, 2047-2054, doi:10.1083/jcb.101.6.2047 (1985).

Eukaryotic cell lines

Policy information about cell lines

Cell line source(s)

HEK293T cells (ICLC)
HeLa cells (ATCC)
CaCo-2 cells (ATCC)

Authentication

At each batch freezing all cell lines were authenticated by STR profiling (StemElite ID System, Promega)

Mycoplasma contamination

mycoplasma test was performed at each batch freezing using PCR and biochemical test (MycoAlert, Lonza)

Commonly misidentified lines
(See ICLAC register)

No commonly misidentified cell lines were used.
