# Supplementary material for: Clathrin light chain A drives selective myosin VI recruitment to clathrin-coated pits under membrane tension
Source: Nat Commun. 2019 Oct 31;10:4974. doi: 10.1038/s41467-019-12855-6 (PMC6823378; doi:10.1038/s41467-019-12855-6)
Supplement: Supplementary file 2 — Supplementary Information [file 41467_2019_12855_MOESM2_ESM.pdf]

Clathrin Light Chain A Drives Selective Myosin VI Recruitment to Clathrin-Coated Pits Under Membrane Tension

Biancospino et al.

Supplementary Figure legends

Supplementary Figure 1. Depletion of CLCb increases the recruitment of myosin VI_{long} to CCPs. (**a**) Left panel, the C-terminal part of CHC is required to mediate the interaction with myosin VI_{long}. Top, scheme of the different GFP-tagged clathrin heavy chain constructs used in the experiment together with their ability to interact with myosin $VI^{998-1131}$; bottom, pulldown with GST-myosin $VI^{998-1131}$ from long and short isoforms were incubated with lysates from HEK293T transfected either with the indicated constructs. Bound proteins were resolved in SDS-PAGE and analysed by immunoblot using anti-clathrin heavy chain antibody (clone 23). Pale-green arrows highlight the position of GFP-CHC fusion proteins. Asterisk marks the position of the endogenous CHC protein. Ponceau detects equal loading of GST proteins. Right panel, purified clathrin heavy chain and myosin VI do not interact *in vitro*. Top, scheme of the different GST-tagged CHC constructs used in the experiment together with their ability to interact with myosin VI_{long} (aa 998-1131); bottom, bacterially purified GST-tagged CHC proteins (2 μM) were incubated with increasing indicated amount of purified myosin VI fragment. Bound proteins were resolved in SDS-PAGE and stained with Coomassie. (**b**) Immunofluorescence analysis of HeLa cells transfected with GFP-myosin VI long or short isoform and depleted of CLCa or CLCb by siRNA oligos. Red, CHC; green, GFP- myosin VI; white, co-localizing pixels. Scale bars, 10μm. Magnification, 10x10 μm (**c**) Bottom panel, quantification of the co-localization (showed in **b**) using Manders' coefficient. Graph represents average of 3 independent experiments (about 450 cells analyzed), error bars represent s.d. * $P<0.05$ ** $P<0.01$ by two-tailed T-test. Right panel, IB of lysates from the same transfected cells shows that CLCs are efficiently depleted. IB as indicated.

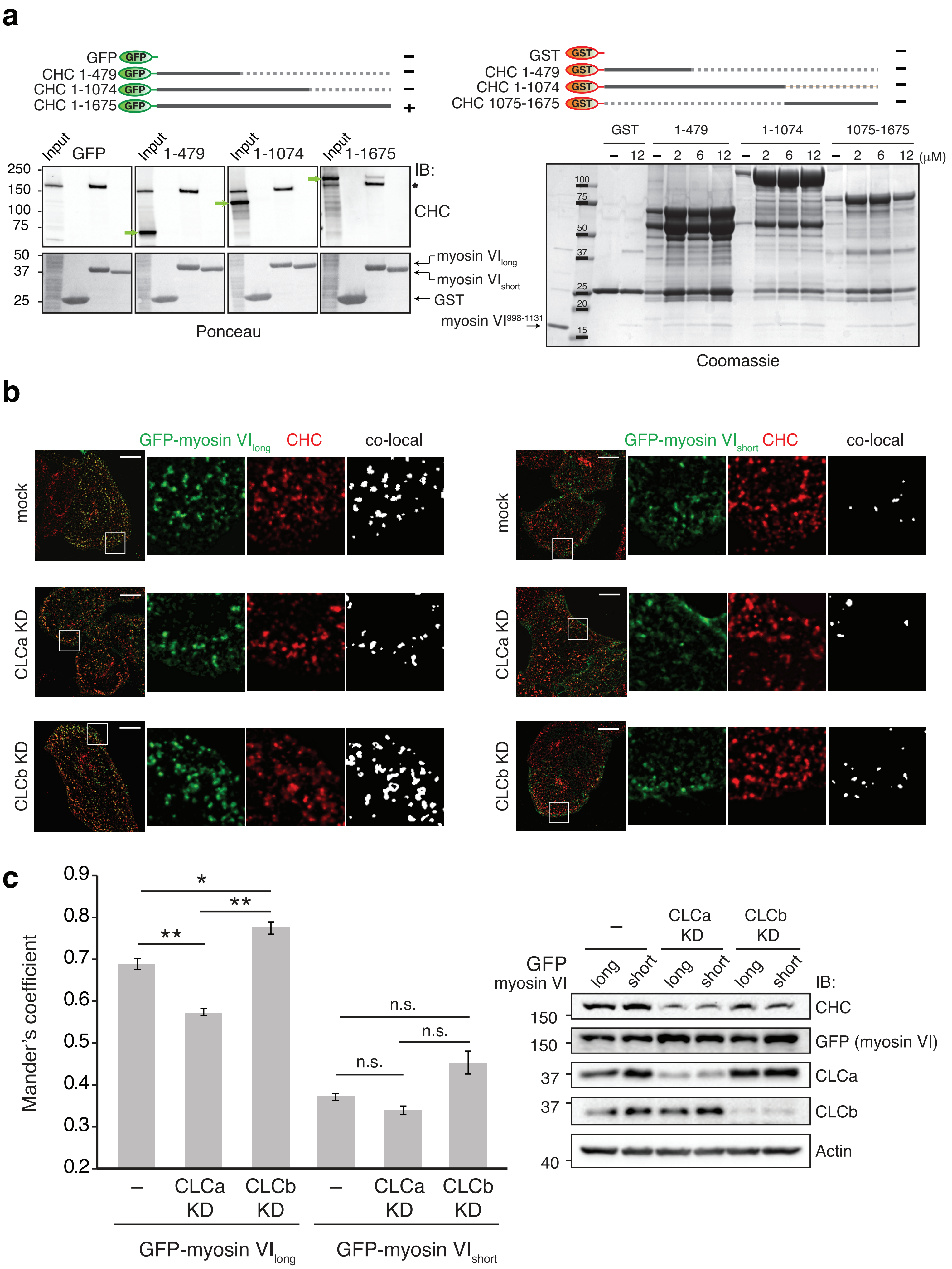

Supplementary Figure 1

Supplementary Figure 2. CLCa is a direct and stable binding partner of myosin VI$_{long}$. (**a**) Flow chart of the preparation of clathrin cages which selectively contain either CLCa or CLCb required for the co-sedimentation experiment shown in **Fig. 1d**. (**b**) IB analysis of purified native or stripped for CLCs clathrin triskelia performed with the indicated antibodies. (**c**) Representative transmission electron micrographs of negatively stained native and CHC-only clathrin and CLCa- or CLCb-reconstituted cages obtained after dialysis with buffer A. Scale-bars: 500nm. (**d**) Analytical size exclusion chromatography of 50 μM CLCa$^{47-97}$ peptide (green line), 50 μM myosin VI$^{998-1131}$ fragment (red line) or the complex formed by the two polypeptides incubated at 1:1 ratio for 15 min at 4ºC (blue line). Note that CLCa$^{47-97}$ peptide migrates aberrantly at 20 KDa. (**e**) Pulldown assay with GST-CLCa and CLCb full-length and the indicated fragments of CLCa immobilized on glutathione sepharose beads and incubated with the purified fragment spanning aa 998-1131 of myosin VI$_{long}$. After washes, bound proteins were eluted in Laemmli-buffer, resolved through SDS-PAGE, and stained with Coomassie.

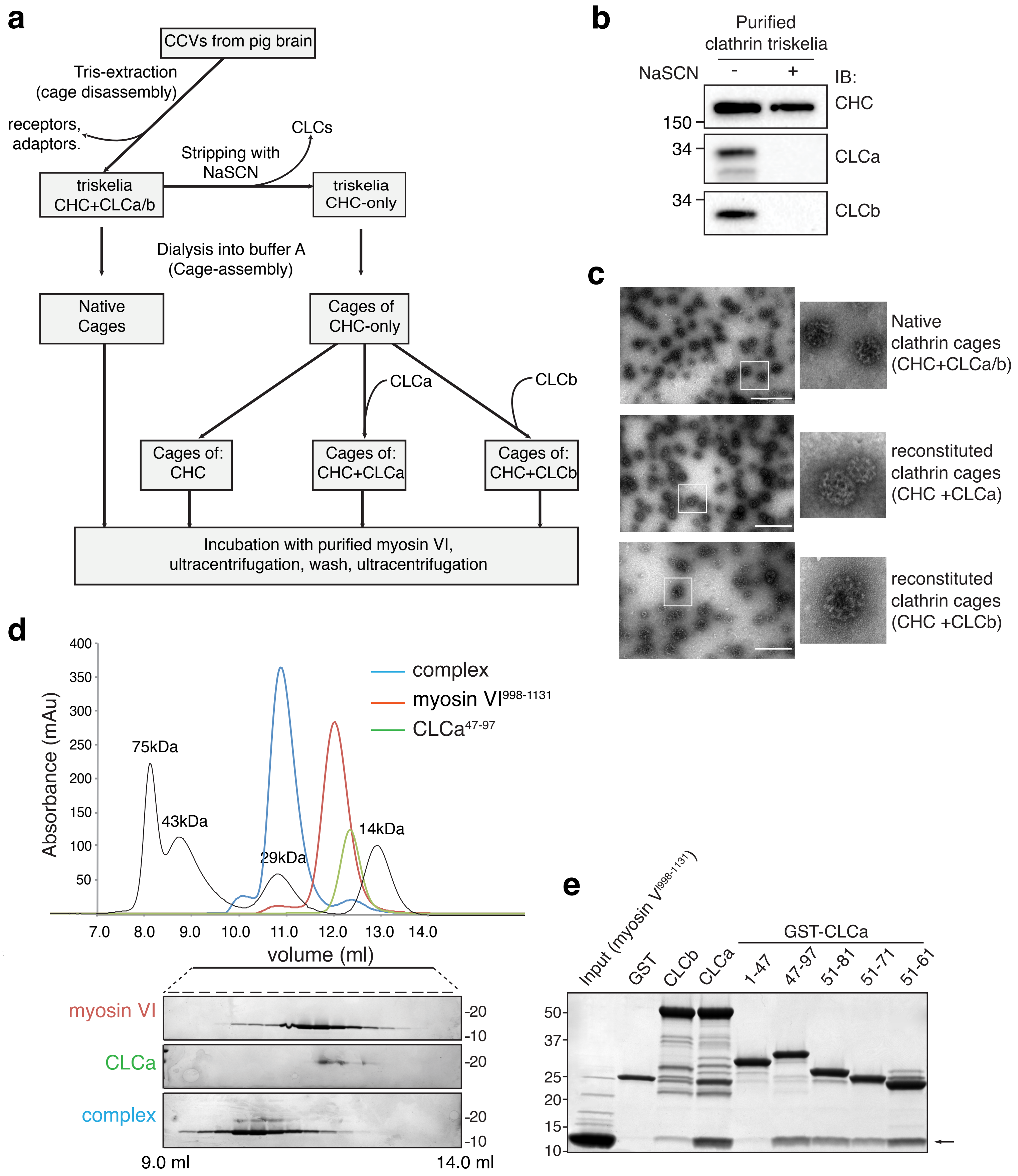

Supplementary Figure 2

Supplementary Figure 3. Chemical shift perturbation (CSP) analysis of myosin VI binding to CLCa. The CSP value is plotted for each amide along the amino acid sequence, calculated according to the definition $CSP = [(0.2\Delta\delta N)^2 + (\Delta\delta H)^2]^{1/2}$ where $\Delta\delta N$ and $\Delta\delta H$ represent chemical shift differences of amide nitrogen and proton atoms for each residue following CLCa addition. A diagram is included below the plot to illustrate the location of the myosin VI helices, with the isoform-specific $\alpha 2$ in orange and the MyUb domain helices in blue. The mean value and 1 standard deviation (SD) above the mean are indicated by an orange and red line respectively. # indicates proline residues which lack amide protons and are thus excluded from this analysis. Residues with CSP values one SD above the mean are labeled.

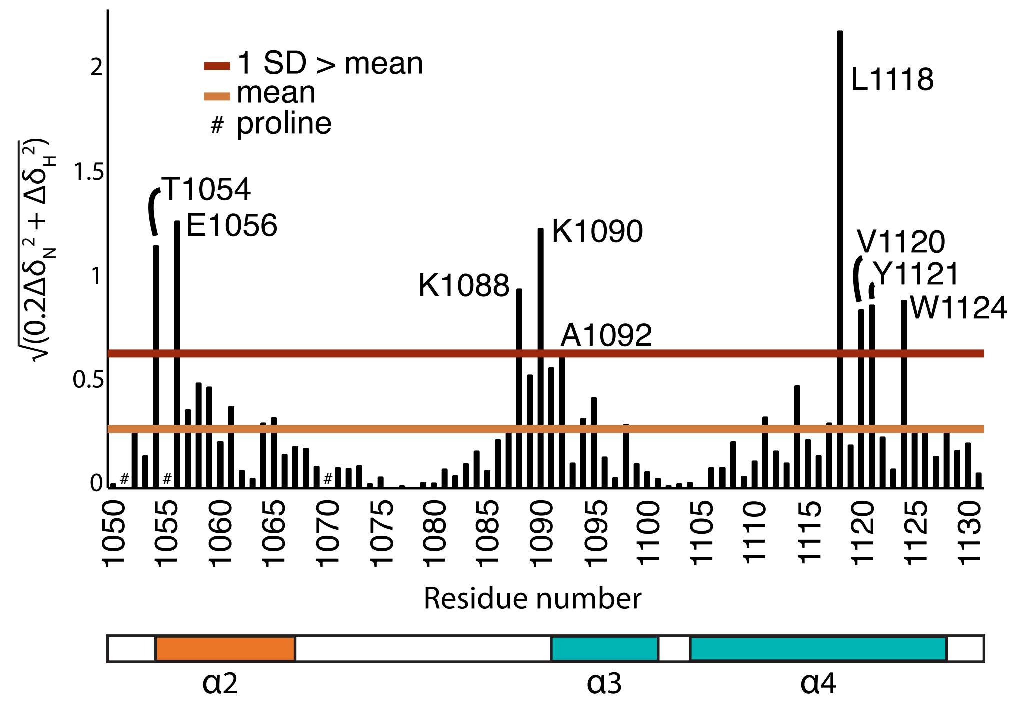

Supplementary Figure 3

Supplementary Figure 4. HNCACB spectrum collected on 0.4 mM ^{13}C, ^{15}N labeled CLCa 46-61 and equimolar unlabeled myosin VI. This spectrum was recorded at 10°C and at 850 MHz. A large dynamic range is apparent with strong signals at the terminal ends of this CLCa peptide and weaker signal for residues A51 – G57. Residues L55 and D56 are missing from this spectrum but apparent in NOESY experiments, including that presented in Supplementary Figure 5. In this experiment, $C\beta$ (red) and $C\alpha$ (black) signals are 180° different in phase.

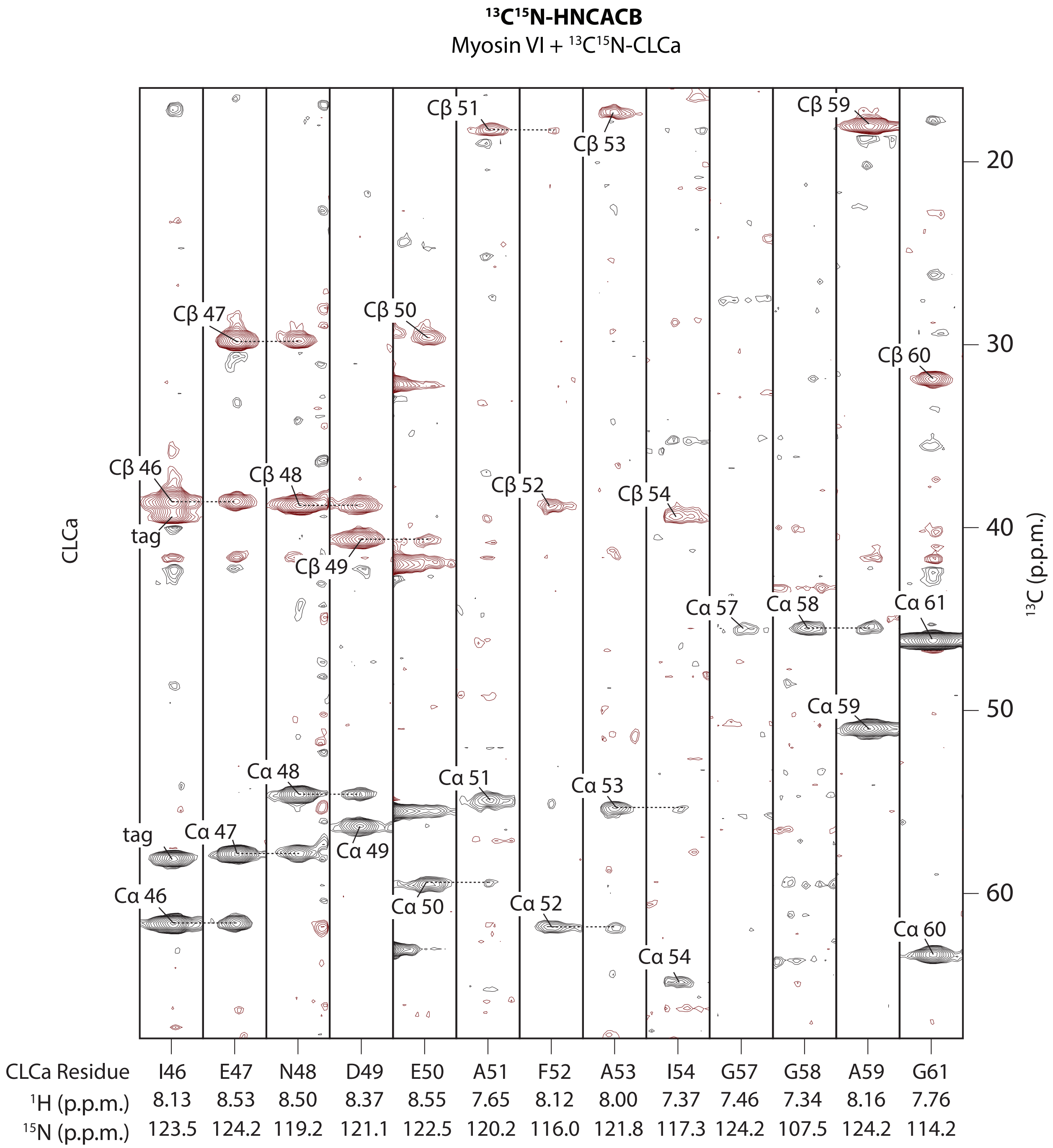

Supplementary Figure 4

Supplementary Figure 5. Selected regions from a ^{15}N-dispersed NOESY experiment acquired with a 120 ms mixing time on 0.4 mM ^{13}C, ^{15}N labeled CLCa 46-61 and equimolar unlabeled myosin VI. This spectrum was acquired at 10°C and at 850 MHz. The top panel shows Hα to HN NOE interactions while the bottom panel displays interactions between amide protons and to the aromatic sidechain of F52. With the exception of R1117, all NOEs are intramolecular.

^{15}N-NOESY

Myosin VI + ^{13}C^{15}N-CLCa

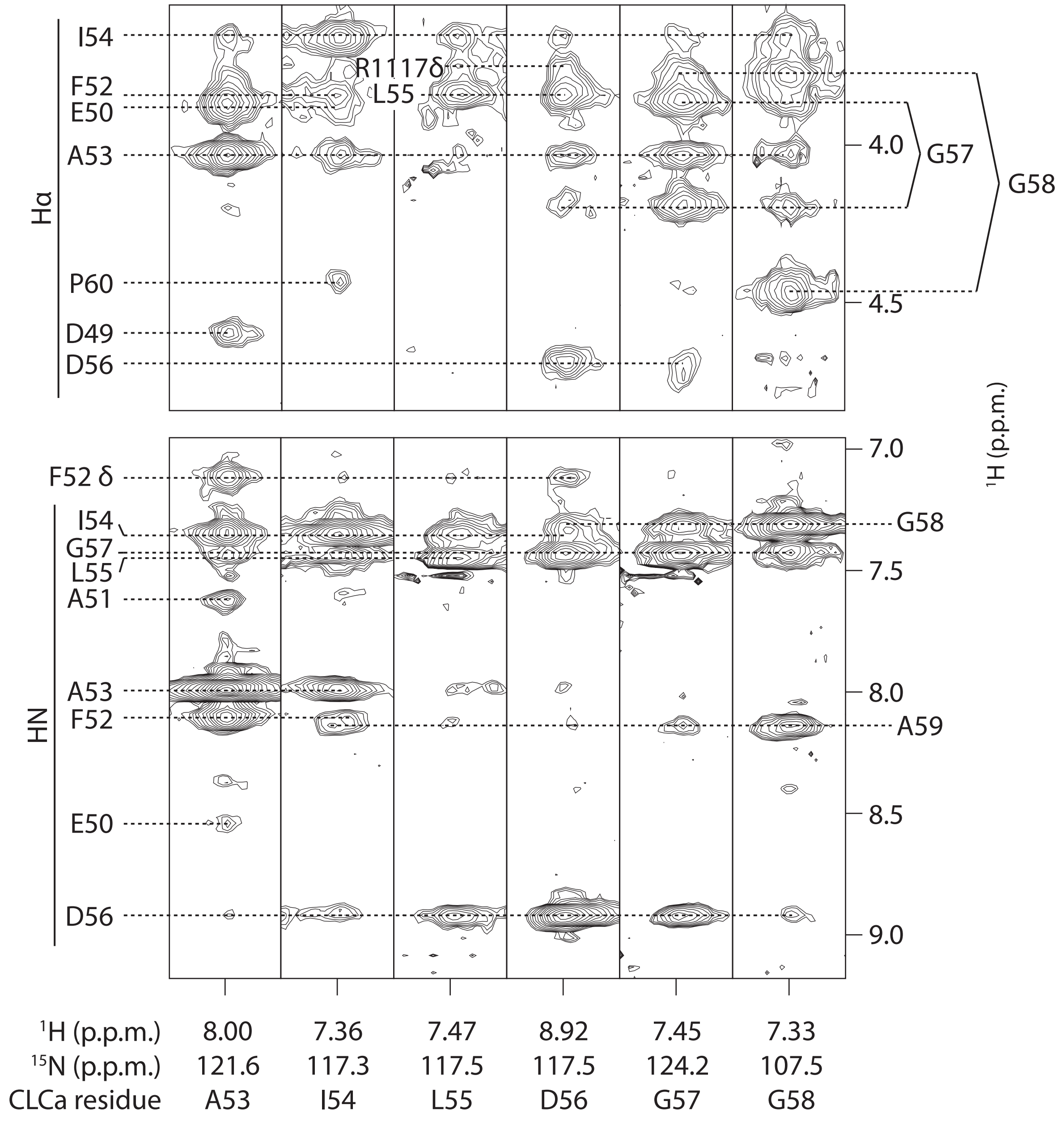

Supplementary Figure 5

Supplementary Figure 6. Summary of selected NMR data indicating a helical configuration for CLCa$^{47\text{-}54}$. The amino acid sequence of CLCa$^{46\text{-}61}$ is diagrammed with experimental NMR data. The alpha carbon (Cα) and carbonyl carbon (C') chemical shift indices (CSIs) are indicated as either positive or negative corresponding to chemical shift values larger or smaller than that expected for a random coil, respectively; differences within 0.7 ppm for Cα or 0.5 ppm for C' are denoted 0 (random coil values as published by Wishart & Sykes, 1994). Bars connecting two residues indicate that an NOE interaction was detected between the α and β hydrogens [NOE αβ(i,i+3)] or the α and backbone amide hydrogen (NOE αN as indicated).

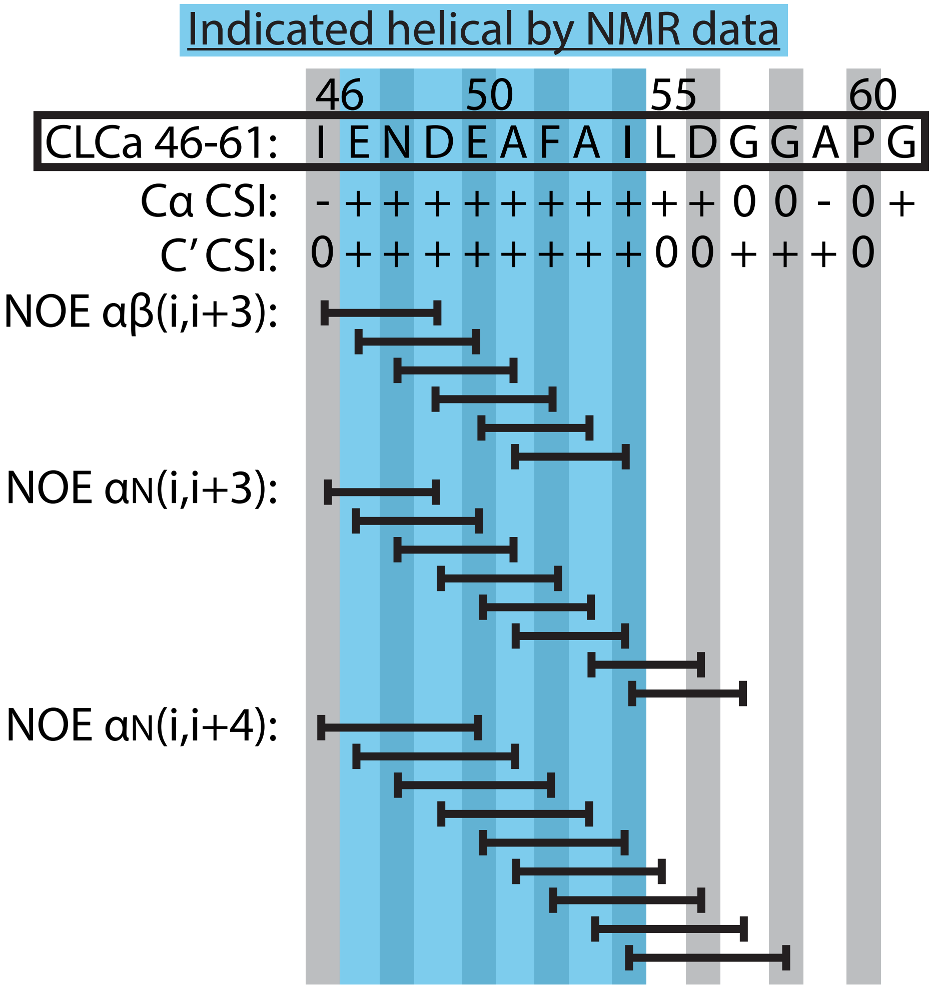

Supplementary Figure 6

Supplementary Figure 7. (**a**) Selected regions from a ^{13}C half-filtered NOESY experiment acquired on 0.36 mM ^{13}C, ^{15}N-labeled myosin VI and equimolar unlabeled CLCa showing intermolecular NOE interactions between myosin VI residues M1058, A1059, and M1062 and various residues from CLCa. 'D' indicates a diagonal intramolecular breakthrough peak. (**b**) As in **a**, but with regions selected to show intermolecular NOE interactions involving myosin VI P1055. (**c**) As in **a**, but with regions selected to highlight intermolecular NOE interactions involving myosin VI V1120 and Y1121. (**d**) Selected regions from a ^{13}C half-filtered NOESY experiment highlighting NOE interactions between CLCa residues I46, E47, and E50 and myosin VI W1124. This experiment was acquired on 0.4 mM ^{13}C, ^{15}N labeled CLCa 46-61 and equimolar unlabeled myosin VI. (**e**) As in **a**, but with regions selected to show intermolecular NOE interactions involving myosin VI W1124. (**f**) Selected regions from a ^{15}N-dispersed NOESY experiment acquired on 0.36 mM ^{15}N, ^{13}C labeled myosin VI 1050-1131 and equimolar unlabeled CLCa 46-61 to demonstrate intermolecular NOE interactions involving the W1124 amide protons. Intramolecular and intermolecular interactions are labeled in grey and black respectively.

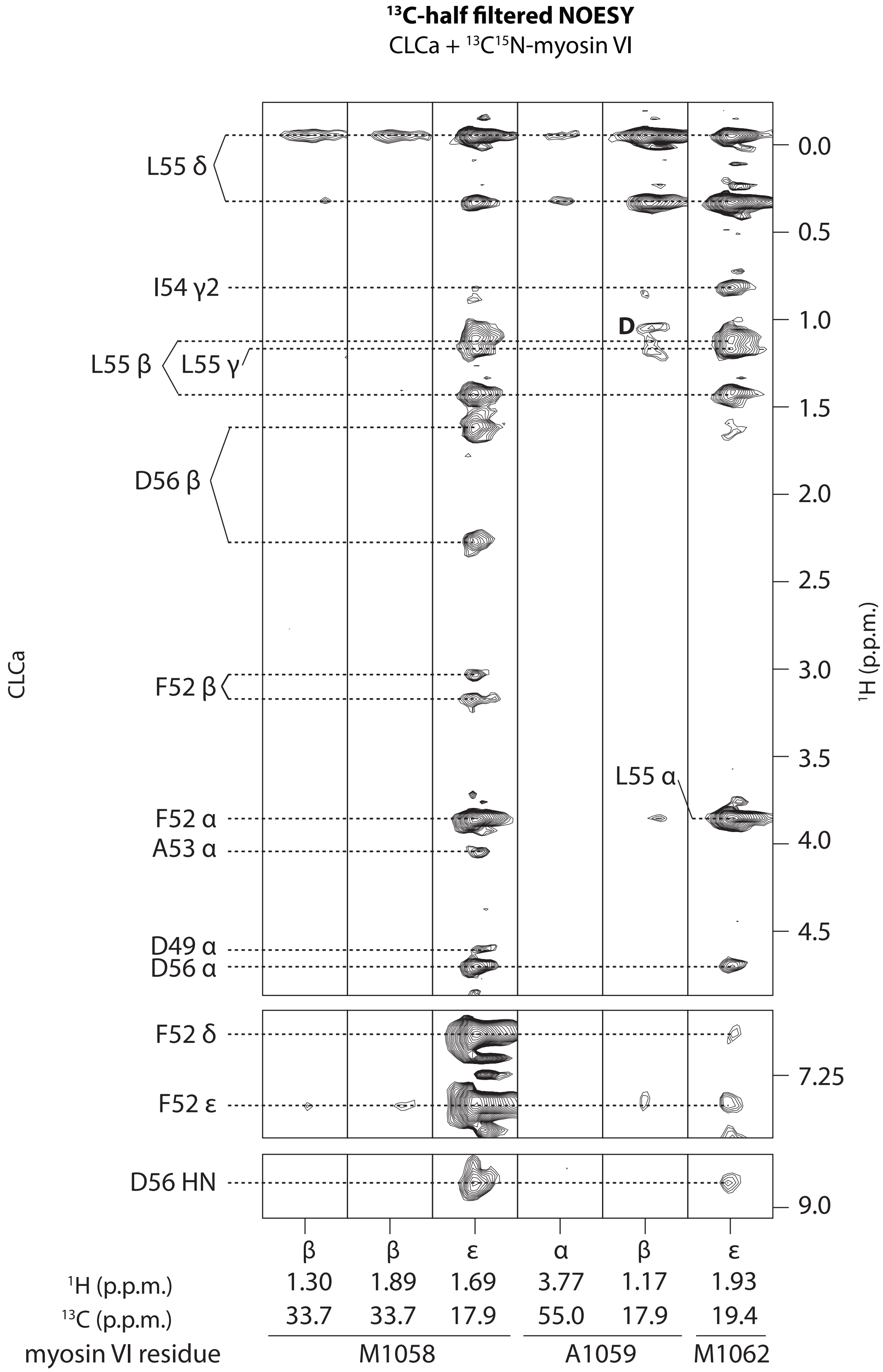

Supplementary Figure 7a

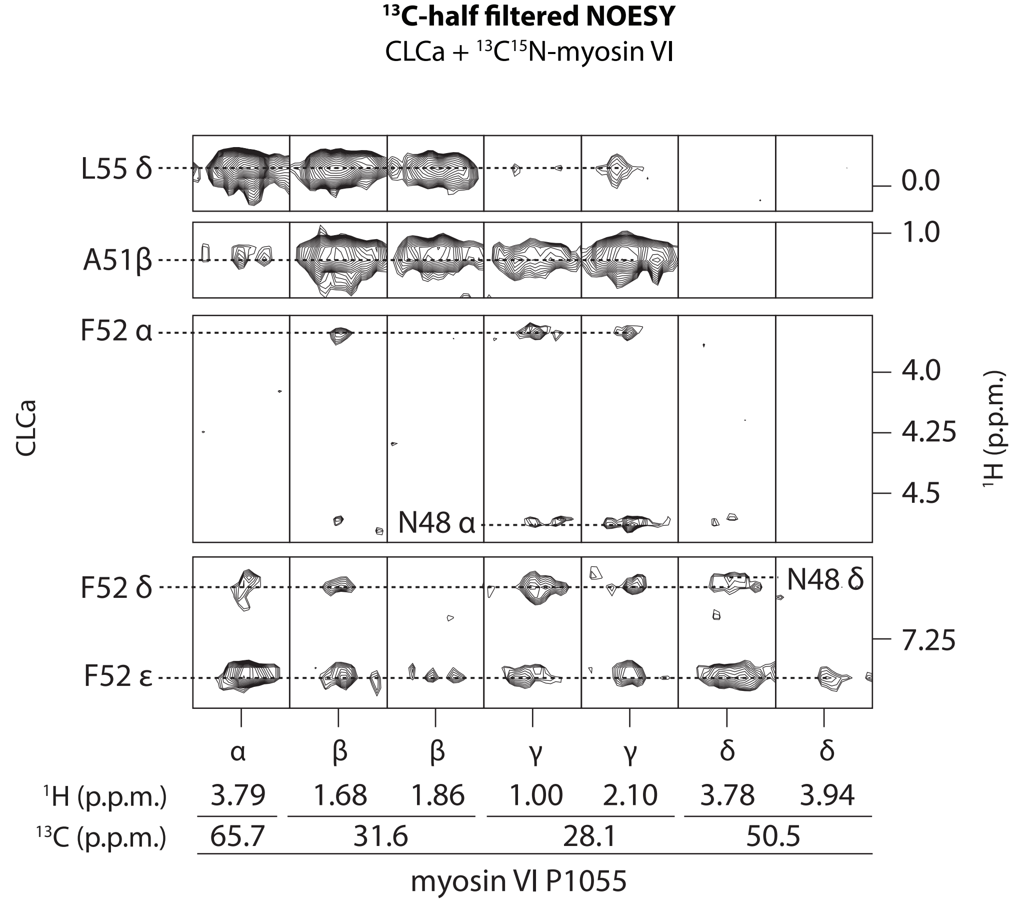

Supplementary Figure 7b

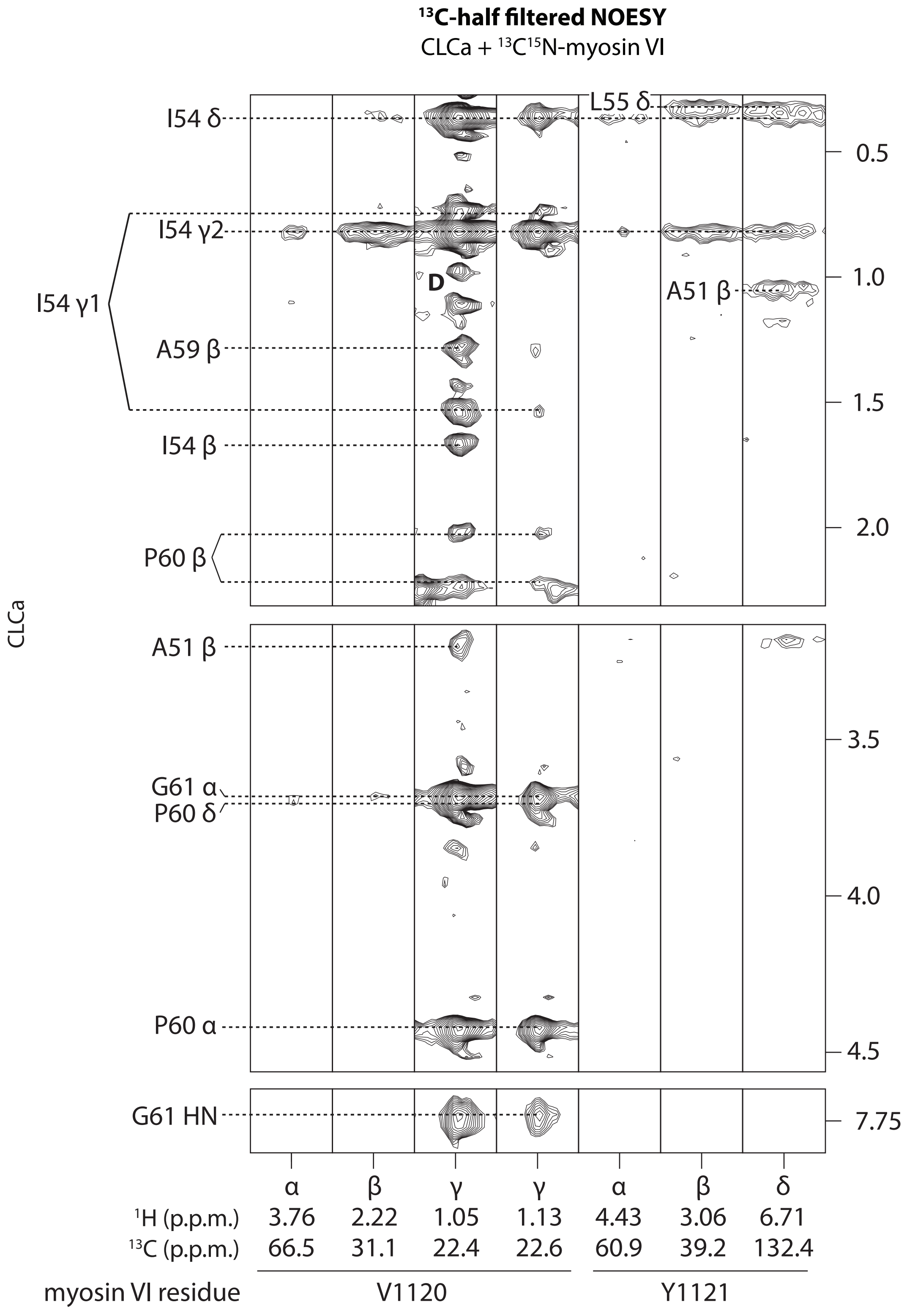

Supplementary Figure 7c

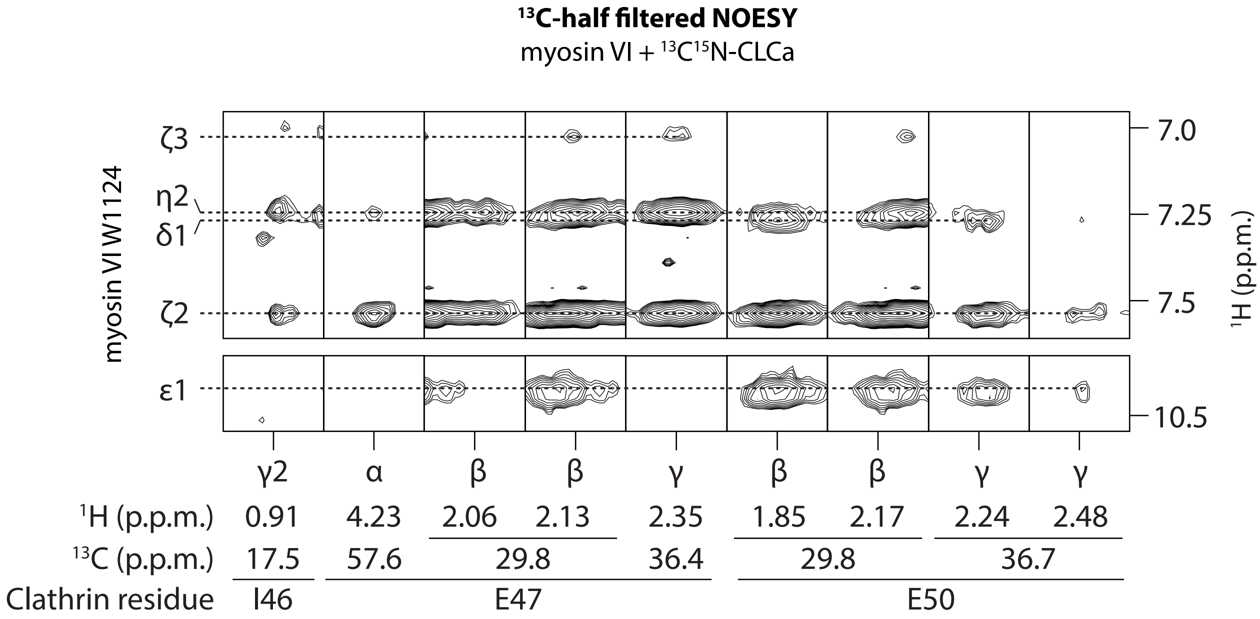

Supplementary Figure 7d

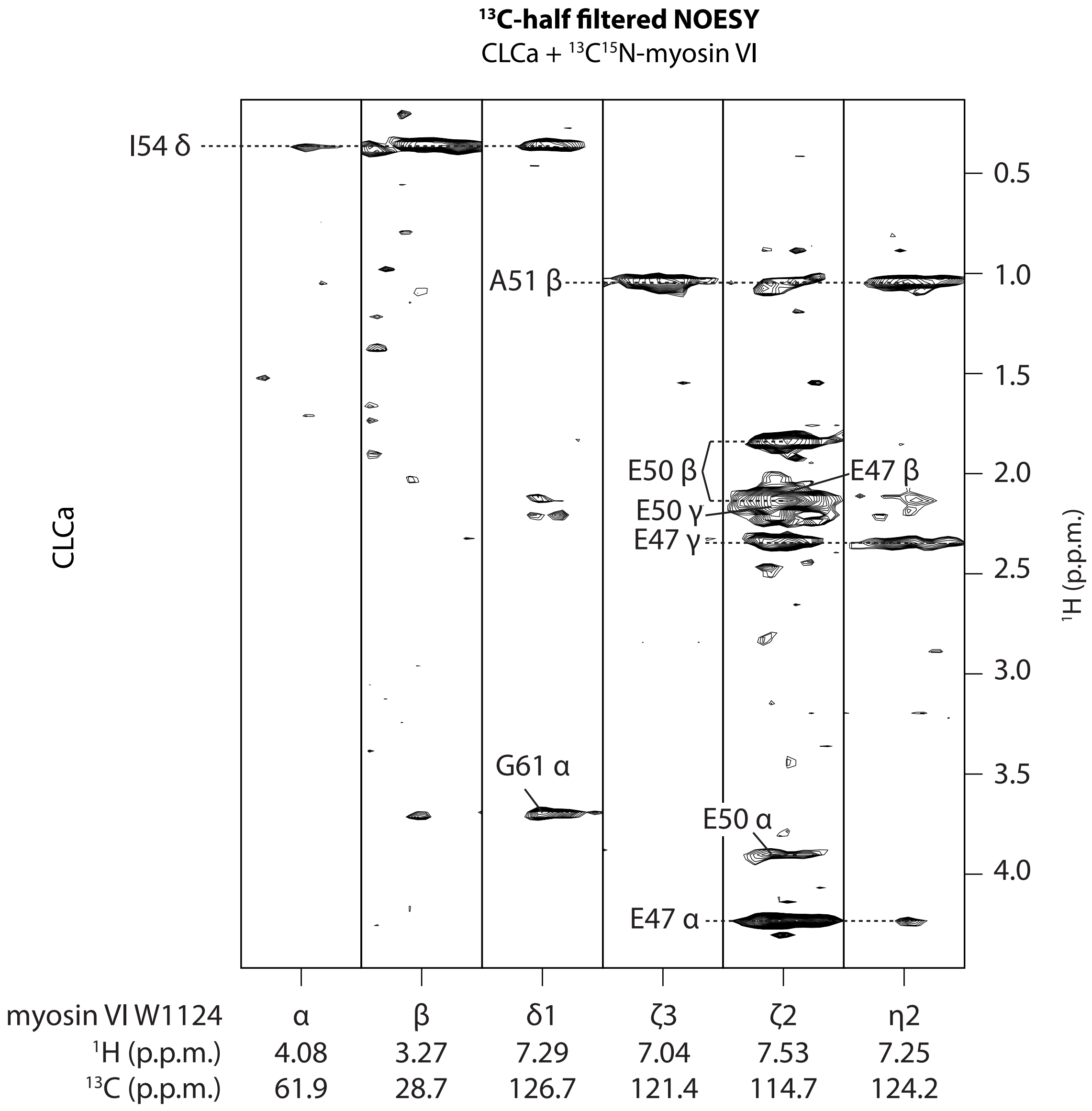

Supplementary Figure 7e

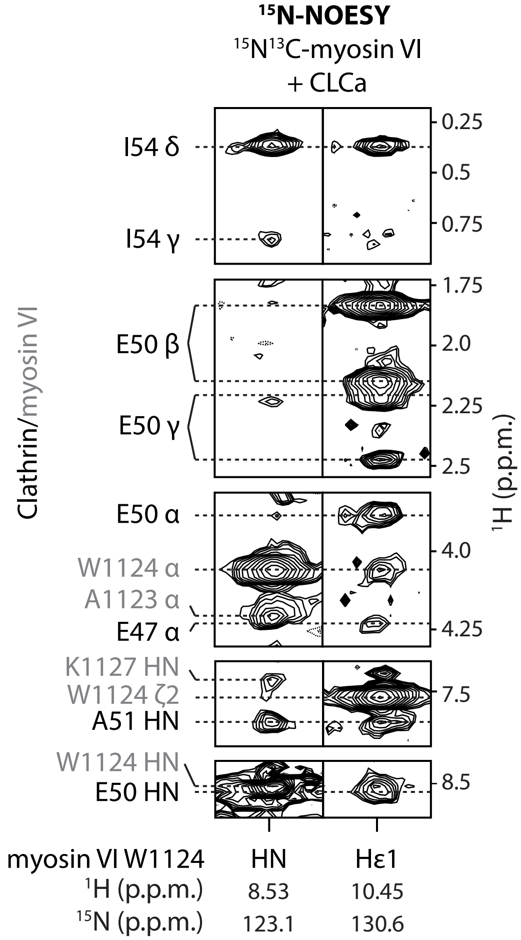

Supplementary Figure 7f

Supplementary Figure 8. Selected regions from a 3D ^{13}C half-filtered NOESY experiment acquired on 0.4 mM ^{13}C, ^{15}N labeled CLCa$^{46\text{-}61}$ and equimolar unlabeled myosin VI$^{1050\text{-}1131}$. NOEs to CLCa from the α2 or α4 helices of myosin VI are labeled in orange or cyan, respectively; 'D' indicates a breakthrough intramolecular diagonal peak.

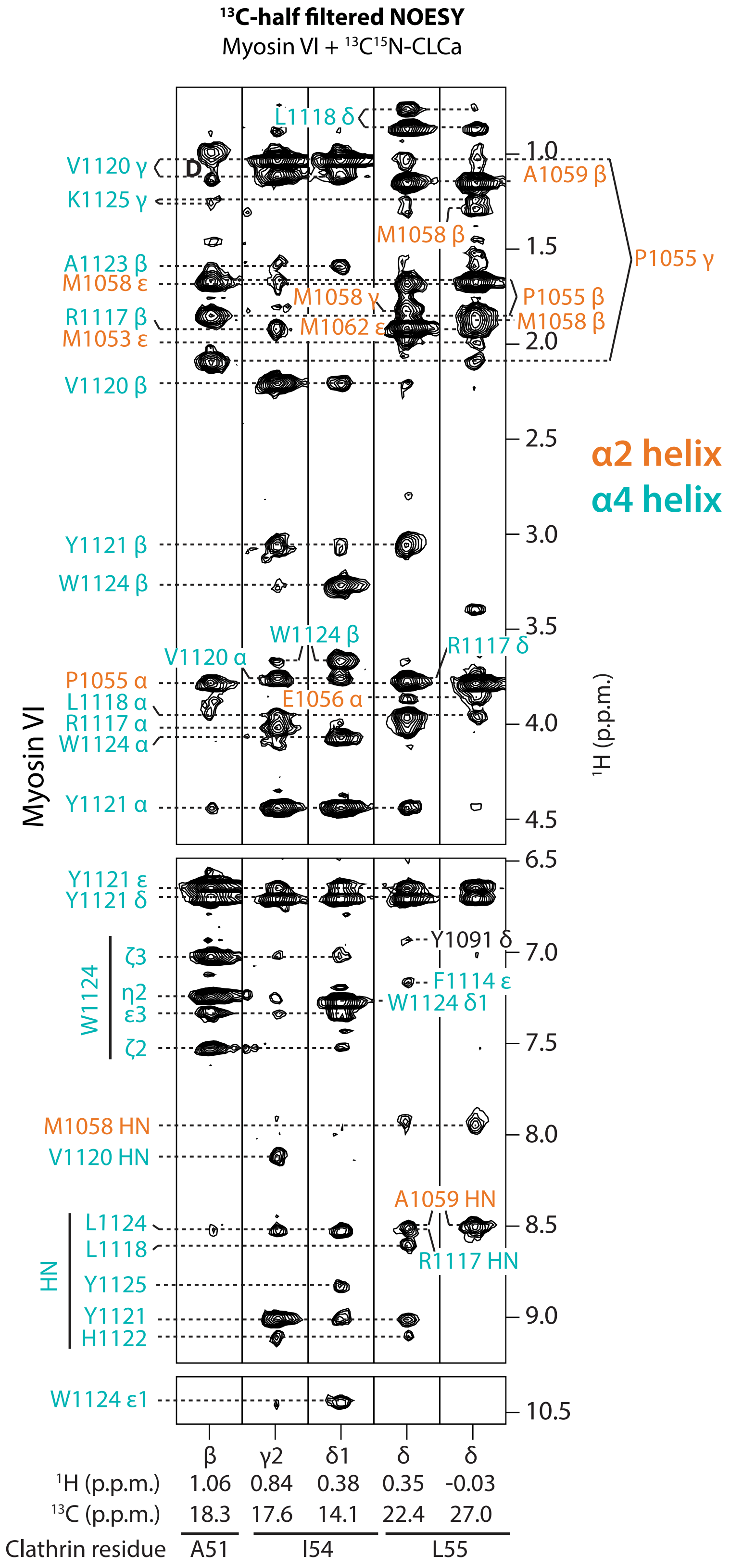

Supplementary Figure 8

Supplementary Figure 9. Characterization of CLCa:myosin VI$_{long}$ interaction. (**a**) Pulldown assay with cleaved and purified CLCa full-length and GST-tagged myosin VI spanning aa. 998-1131 or aa. 998-1122 from myosin VI$_{long}$ or myosin VI$_{short}$ (described in the upper panel). Due to the similar mw of CLCa and GST-myosin constructs detection was performed with IB using anti-clathrin light chain antibody (X16). Ponceau detects equal loading of GST proteins. (**b**) FP assay with myosin VI Y1121A mutant. Fluorescein-tagged CLCa$^{46-61}$ was incubated with increasing amount of either myosin VI$^{1050-1131}$ wild-type or Y1121A mutant. Plots are representative of a single experiment with dissociation constants reported above. Partial loss of structural integrity may contribute to the reduction in binding shown by myosin VI mutant as Y1121 also interacts intramolecularly with the isoform-specific helix.

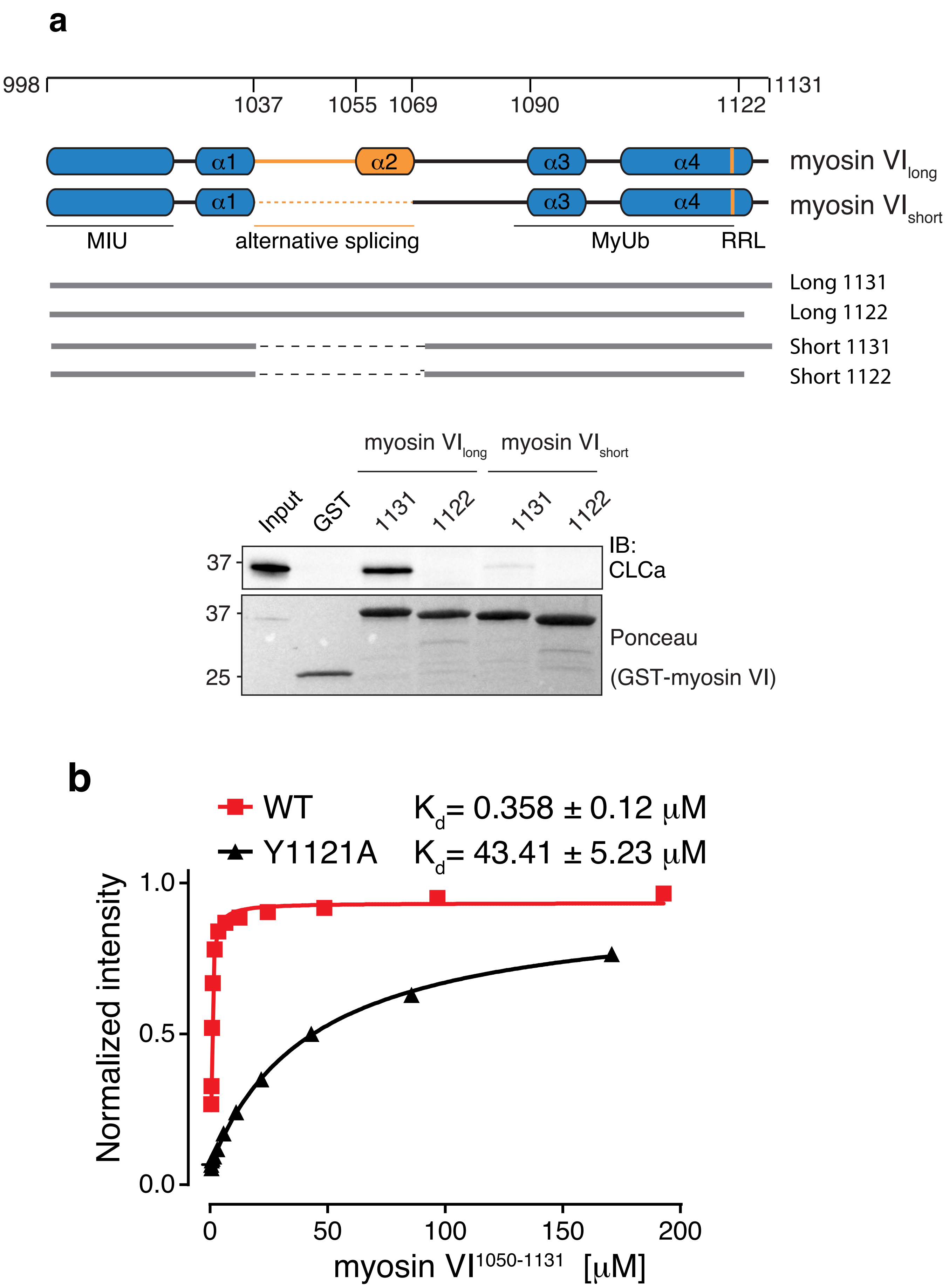

Supplementary Figure 9

Supplementary Figure 10. Apico-basal polarity is properly rescued and preserved in the I54D mutant cysts. (**a**) RT-PCR analysis of cDNA obtained from human and murine intestine and kidneys. Epithelial tissues from both organisms uniquely express myosin VI_{long}. (**b**) Confocal micrographs of kidney's cryosections from adult mice. Myosin VI_{long} predominantly localized at the apical surface towards the lumen of the organs, at the basis of the brush-border; phalloidin staining identifies distal tip of microvilli. Scale bar: 6 μm. (**c**) RT-PCR analysis of cDNA obtained from Caco-2 cells grown in non-polarized sparse conditions or after 5 days of confluency in 2D or 7 days in 3D (fully formed cysts) conditions. A clear switch towards the myosin VI_{long} isoform occurs when Caco-2 are grown as epithelial polarized monolayer or upon cyst formation. (**d**) Representative confocal micrographs of Caco-2 cysts. Myosin VI mainly localize at the apical terminal web region as highlighted by the co-localization with the apical marker occludin. Scale bars: 25 μm. (**e**) TEM of polarized Caco-2 cysts. Note the establishment of an apical-basal polarity in the cyst, represented by the extensive microvilli formation at the lumen and the presence of junctional complexes (indicated by arrowheads). Scale bars: 2 μm. (**f**) Representative confocal micrographs of CLCa wild-type and I54D localization in Caco-2 cysts. Staining with the indicated apical-basal polarity markers are shown. Scale bars: 100 μm.

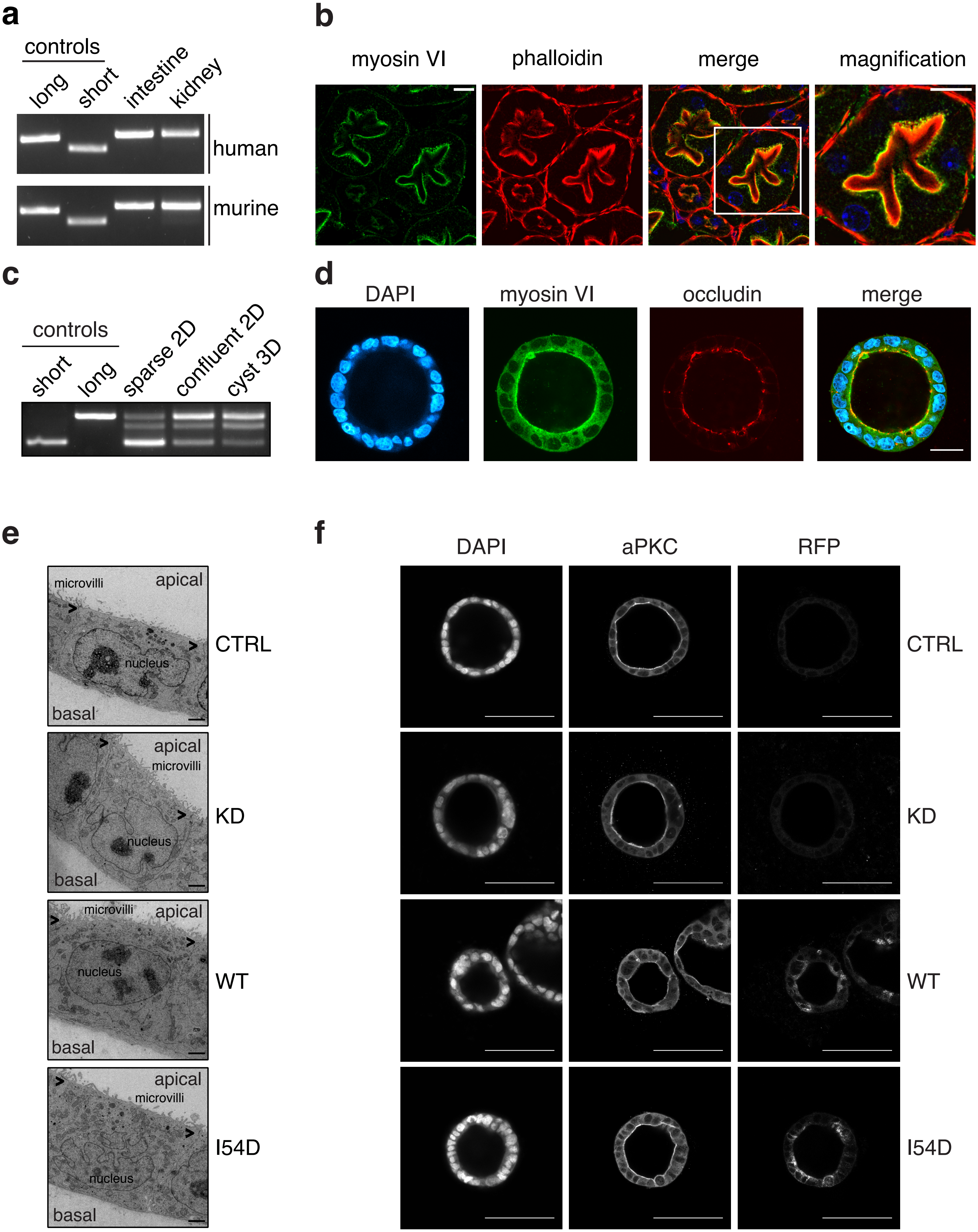

Supplementary Figure 10

Supplementary Figure 11. Impairment of CLCa:myosin VI interaction affects the dynamics of clathrin pits in polarized cysts. (**a**) qPCR assessing endogenous CLCa or CLCb depletion in Caco-2 cells. Knockdown cells (Caco-2, Caco-2 RFP-rat-CLCa wild-type or Caco-2 RFP-rat-CLCa I54D) generated by siRNA oligos transfection (t0) were embedded in Matrigel and cultured for 7 days to allow cysts formation (t7). Endogenous CLCa/b were efficiently depleted during the entire process. (**b**) qPCR assessing endogenous CHC upon CLCs depletion. Samples are as in a. (**c**) Hela cells knockdown for CLCs generated by siRNA oligos transfection are lysed after 48h. For oligos sequence, see Methods. IB as indicated. Confirming the effect see in Caco-2 cells (Fig. 5a), CHC level decreases upon CLCa depletion with both oligos while CLCb depletion does not affect CHC expression. (**d**) Morphometrical analysis of the distribution of clathrin-coated structures at the apical surface of polarized Caco-2 cysts. Pits were classified as shallow, omega/constricted or elongated according to their morphology. Clathrin-coated vesicles were excluded from the analysis. Data are expressed as: right axis (bars), number of pits normalized to PM profile length (µm), ± SEM; left axis (scattered dot plot), average number of coated structures per cells. Each dot color indicates an independent experiment. Total cellular profiles (N) analyzed for CTRL, KD, rescue WT and I54D were respectively 125, 98, 94 and 101. For statistical analysis the number of pits from 3 independent experiments were pooled together and P-values were calculated using Oneway ANOVA. *, $P < 0.05$; ** $P < 0.01$ (vs. control). (**f**) ITC experiments with the indicated proteins. The integrated heat plot and the raw plot are reported. Equilibrium dissociation constants (KD) obtained by the fitting are indicated below. Relevant ITC measurements are reported in Table 2.

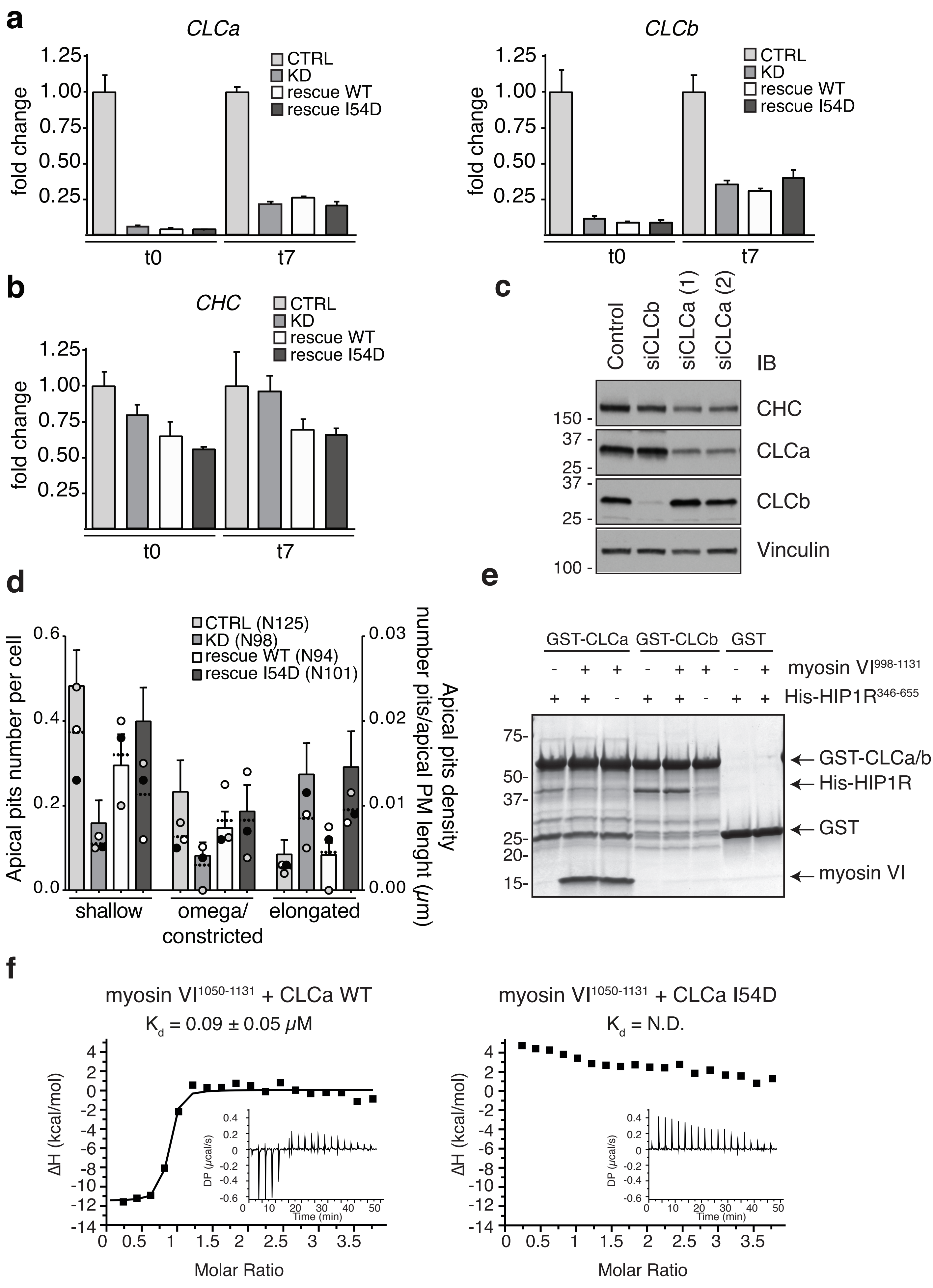

Supplementary Figure 11

Supplementary Figure 12. Ca^{2+} mobilizes CLCa and myosin VI improving their interaction. Co-immunoprecipitation of endogenous CLCa and myosin VI in presence of high Ca^{2+} concentration. Endogenous proteins were immunoprecipitated from lysates of polarized Caco-2 cells (5 days after confluency) obtained in JS buffer (-) or in JS buffer with 2 mM $CaCl_2$ (+). IP was performed either with anti-myosin VI antibody (1295) (**a**) or with anti- CLCa antibody (X16) (**b**). IB as indicated. Note that an improved binding in high calcium condition is clearly visible using both antibodies. Values reported below each blot are normalized for the immunoprecipitated proteins (myosin in the upper gel, CLCa in the lower gel) and represent the fold change relative to the sample lysed in JS (-). Experiments are repeated twice with similar results.

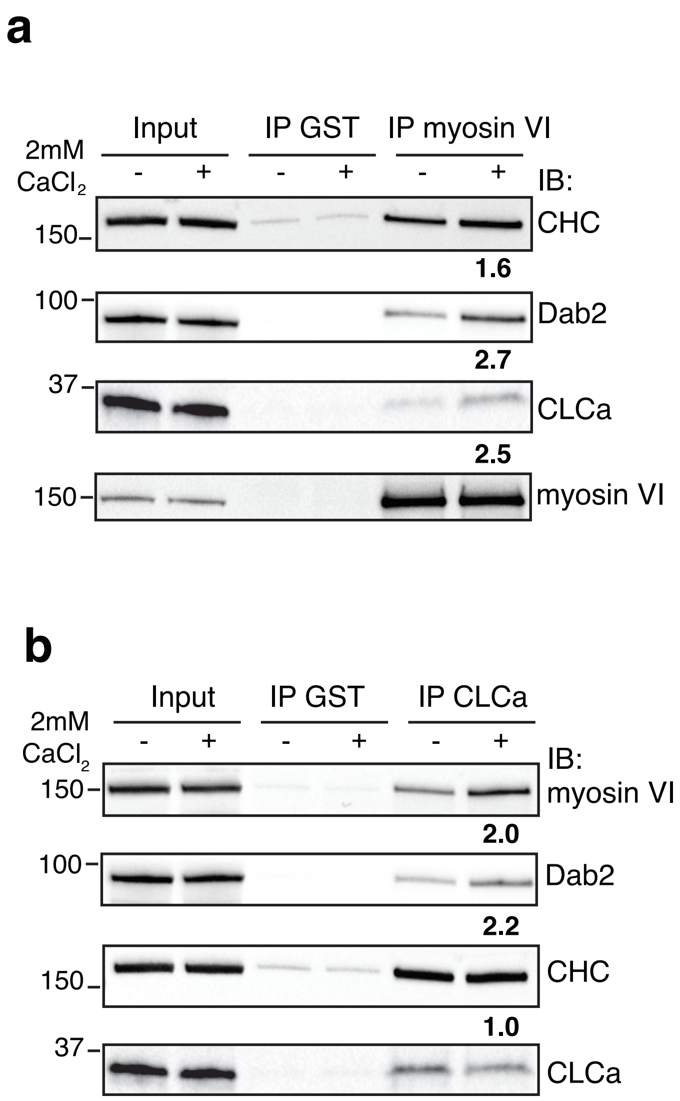

Supplementary Figure 12

Supplementary Table 1. List of used primers.

	Forward (5'-3')	Reverse (5'-3')
hCLCa Nterm	CCGGAATTCATGGCTGAGCTGGATCCGTTCGG	
hCLCa aa50	CCGGAATTCGCCTTCGCCATCCTGGACGGC	
CLCa 47-97	TAGAATTCAACGACGAGGCCTTCGCCATC	ATACTCGAGTCAAATAGCTGCATAACTGTCTGTTG
CLCa STOP157	CGAGCAGCTACAGAAAACATGAGCAAACAACAGGGCAGCAG	CTGCTGCCCTGTTGTTTGCTCATGTTTTCTGTAGCTGCTCG
CLCa STOP97	GACGTTATGCAGCTATTTGACAAGTGGATCGATTGCAG	CTGCAATCGATCCACTTGTCAAATAGCTGCATAACGTC
CLCa STOP47	GATTGCGGGCATCGAGTGAGACGAGGCCTTCGCCATC	GATGGCGAAGGCCTCGTCTCACTCGATGCCCGCAATC
hCLCb	GGAATTCATGGCTGATGACTTTGGCTTCTTCTCG	CGGCTCGAGGGATCCCTAGCGGGACAGTGGCGTCTG
hCLCa 46-61 deletion	PHOS-GCCCGCAATCTCGCTCTCTTGCTG	PHOS-CCCCAGCCGCACGGCGAGC
rCLCa I54D	GACGAGGCCTTCGCCGACCTGGACGGCGGCG	CGCCGCCGTCCAGGTCGGCGAAGGCCTCGTC
hCLCa I54A	CCGCCGTCCAGAGCGGCGAAGGCCTCGT	ACGAGGCCTTCGCCGCTCTGGACGGCGG
hCLCa I54D	ACGAGGCCTTCGCCGACCTGGACGGCGG	CCGCCGTCCAGGTCGGCGAAGGCCTCGT
hCLCa 61+Tyr+STOP	CGGCGCCCCCGGGTATTAGCAGCCGCACG	CGTGCGGCTGCTAATACCCGGGGGCGCCG
hCLCa G61-STOP	CGGCGGCGCCCCCTAGCCGCAGCCACAC	GTGTGGCTGCGGCTAGGGGGCGCCGCCG
hCLCa G71-STOP	CGGCGAGCCGCCGGGATAACCGGATGCTGTTGA	TCAACAGCATCCGGTTATCCCGGCGGCTCGCCG
hCLCa aa80		CCGCTCGAGTCAATTCATTACTCCATCAACAGCATCCGG
Myo VI W1124A	GGAGACTAAAAGTGTATCATGCTGCGAAATCTAAGAACAAGAAGAGATGA	TCATCTCTTCTTGTTCTTAGATTTCGCAGCATGATACACTTTTAGTCTCC
Myo VI W1124E	GGAGACTAAAAGTGTATCATGCTGAGAAATCTAAGAACAAGAAGAGATGA	TCATCTCTTCTTGTTCTTAGATTTCTCAGCATGATACACTTTTAGTCTCC

Myo VI M1058A	AGACCCAAAATGACACCGGAACAAGCGGCCAAAGAAATGTCAGAATTTTTG	CAAAAATTCTGACATTTCTTTGGCCGCTTGTTCCGGTGTCATTTTGGGTCT
Myo VI M1058E	AGACCCAAAATGACACCGGAACAAGAGGCCAAAGAAATGTCAGAATTTTTG	CAAAAATTCTGACATTTCTTTGGCCTCTTGTTCCGGTGTCATTTTGGGTCT

Supplementary Table 2. List of used antibodies.

Antibody	Species	Supplier	Code	WB	IF
anti-GFP	Rabbit	Sigma	G1544	1:5000	1:200
RFP-TRAP_A	Llama	Chromotek	rta-20		
anti-myosin VI	Rabbit	Ref. [1]	1296	1:2000	1:400 -1:200*
anti-CHC, clone X22	Mouse	Pierce	MA1-065		1:2000
anti-CHC, clone 23	Mouse	BD bioscience	610499	1:1000	
anti-CLCa	Mouse	Ref. [2]	X16	1:500	
anti-CLCa, CLTA	Rabbit	Proteintech	10852-1-AP	1:500	
anti-CLCb, CLTB	Rabbit	Proteintech	10455-1-AP	1:2000	
anti-Dab2	Mouse	BD bioscience	610464	1:2000	
anti-Hip1R	Rabbit	Sigma	HPA038135	1:1000	
anti-actin	Mouse	Sigma		1:1000	
anti-tubulin	Mouse	Sigma		1:1000	
anti-E-cadherin	Mouse	BD Biosciences	610181		1:100*
anti-Zo-1	Rabbit	Thermo Fisher	40-2200		1:100*
anti-Occludin	Mouse	SCBT	E5		1:100*
Anti-PKC zeta	Rabbit	Santa Cruz	Sc-216		1:100*

anti-His	Mouse	GE Healthcare	27-4710-01	1:1000	
phalloidin TRITC					1:50
anti-Mouse IgG HRP	Goat	Bio-Rad	1721011	1:10000	
anti-Rabbit IgG HRP	Goat	Bio-Rad	1706515	1:10000	
anti-rabbit Alexa647	Donkey	Thermo Fischer	A31571		1:400-1:100*
anti-rabbit Alexa488	Donkey	Thermo Fischer	A21206		1:400-1:100*
anti-mouse Alexa488	Donkey	Thermo Fischer	A21202		1:400-1:100*
anti-rabbit Cy3	Donkey	Jackson Lab	711-165-152		1:800
anti-mouse Cy3	Donkey	Jackson Lab	715-165-150		1:800-1:100*

*the indicated dilution was used for immunofluorescence of Cysts.

1 Wollscheid, H. P. *et al.* Diverse functions of myosin VI elucidated by an isoform-specific alpha-helix domain. *Nat Struct Mol Biol* **23**, 300-308, doi:10.1038/nsmb.3187 (2016).

2 Brodsky, F. M. Clathrin structure characterized with monoclonal antibodies. I. Analysis of multiple antigenic sites. *The Journal of cell biology* **101**, 2047-2054, doi:10.1083/jcb.101.6.2047 (1985).
